# Direct current stimulation of the ear in tinnitus treatment: a double-blind placebo-controlled study

**DOI:** 10.1007/s00405-013-2849-6

**Published:** 2013-12-13

**Authors:** Marzena Mielczarek, Jurek Olszewski

**Affiliations:** Department of Otolaryngology and Laryngological Oncology, Medical University of Lodz, 113 Zeromskiego Street, 90-549 Lodz, Poland

**Keywords:** Tinnitus, Electrical stimulation, Direct current, Placebo

## Abstract

The objectives of the study are assessment of the influence of direct current electrical stimulations of the ear in tinnitus treatment, comparison of the results with placebo group and evaluation of hearing after electrical stimulations. The study comprised 120 tinnitus and sensorineural hearing loss patients (*n* = 184 tinnitus ears). In group one (*n* = 119 tinnitus ears) the authors applied a non-invasive hydrotransmissive electrical stimulation (15) of the ear, in group two (*n* = 65 tinnitus ears)—placebo electrical stimulation. Direct rectangular, positive polarization current was used. The frequency of stimulation was adjusted according to tinnitus frequency. In group two, the authors used similar procedure, but no current was delivered through the active electrode. Evaluation of tinnitus and hearing was conducted. In groups one and two, directly after the treatment, the number of ears with permanent tinnitus decreased considerably. In group one in 40 ears (33.6 %) tinnitus disappeared; in group two, tinnitus disappeared in four ears (6.1 %). After 30 days, statistically significant changes were observed in group one (*p* < 0.05), which were comparable with results returned 90 days later (*p* > 0.05). Changes in group two (after 30 and 90 days) were not significant (*p* > 0.05). The authors recognized audiometric improvement of hearing (in pure tone audiometry). The application of direct current electrical stimulation of the hearing organ, with current frequencies similar to tinnitus frequencies (selective electrical stimulation), was an efficient method in severe tinnitus treatment. We did not observe a harmful effect of direct current on hearing organ.

## Introduction

Subjective tinnitus is defined as a phantom perception of sound with the absence of external stimulation. Regardless tinnitus pattern (acute, chronic, constant, intermittent, pure tone vs noise-like), it can negatively affect the quality of life. Despite intense, advanced research conducted all over the world, the factor directly responsible for subjective tinnitus’ perception is still not clear. It is known that it is a result of a pathological activity in the nervous system, without corresponding mechanical activity in the cochlea [1]. On the basis of functional MRI we know that this perception is not only purely auditory phenomenon but also limbic-related central nervous system areas take part [2].

According to some research, about 10–20 % of the adult population suffers from tinnitus and it probably occurs with similar frequency among children [3]. Since its etiology is unclear and taking into account the heterogeneity of the tinnitus patients’ group, there is still no satisfactory method of treatment. The most accessible and used as a first-line treatment in outpatient clinics is pharmacotherapy, however, there is no European Medicines Agency (EMA)- or Food and Drug Administration (FDA)-approved drug on the market [4]. Furthermore, cognitive-behavioral therapy or different forms of stimulations (acoustic or electrical) have most promising effects [5–7]. Numerous hypotheses on the etiology of tinnitus may suggest that there is no single mechanism of its onset. In many cases cochlea is an ignition site of tinnitus. That is why, in patients with tinnitus and a cochlear hearing loss (tinnitus spectrum often overlaps with the area of hearing loss) electrical stimulation (e.s.) can be applied as a treatment. E.s. gives good effects in inflammation, pain or nervous system disorders’ treatment, improving the blood flow and tropism of tissues [8]. Nevertheless, with reference to the hearing organ, it is used in few clinical centers in the world. According to Latkowski [9], e.s. increases transmission of neurotransmitters in the synapses, as well as it controls their secretion to the synaptic area. Portman et al. [10] state that it modifies the electrical potentials of the hearing organ. According to Watanabe et al. [11] e.s. improves the blood flow in the inner ear and synchronizes spontaneous impulses in the auditory nerve fibers. E.s. was primarily used in the cochlear implantation in The House Ear Institute.

A non-invasive hydrotransmissive procedure was used by Szymiec et al., Konopka et al., Morawiec-Bajda et al. and Mielczarek et al. [12–16]. Szymiec et al. [12] using low frequency stimulation (50–1,600 Hz) via electrode dipped in saline solution in the external auditory meatus, with the other placed on the ipsilateral mastoid, observed improvement in 48 % of cases, which was comparable to Morawiec-Bajda et al.’s [15] results 46.6 %. Kuk et al. [17] attempted to reduce tinnitus, stimulating with a ball-electrode placed at the tympanic membrane. The authors, using different parameters of current (square, sine, triangular current, within a range of frequencies 62–8,000 Hz) adapted individually according to patients’ response to stimulation, obtained improvement in tinnitus in 50 % of the cases. Kozlowski et al. [18] using similar method of e.s., adapted parameters individually (frequencies within 16–8,000 Hz), reported improvement in tinnitus in 44 % of patients.

The aim of the study was to assess the influence of direct current e.s. of the hearing organ in tinnitus treatment adapting the frequency of current according to tinnitus frequency and to compare these effects with placebo group, as well as to evaluate hearing after e.s. in both groups.

## Materials and methods

Study design: a double-blind placebo-controlled study. The study comprised 120 patients suffering from tinnitus and sensorineural hearing loss (*n* = 184 tinnitus ears) divided into two groups. The patients from group one (*n* = 119 tinnitus ears, 80 tinnitus patients, 38 females and 42 males), aged 21–74 years (average 53.5 ± 9.31), were treated with e.s. of the hearing organ. Those from group two (*n* = 65 tinnitus ears, 40 tinnitus patients, 24 females and 16 males), aged 22–76 years (average 56.5 ± 11.2), were subjected to placebo e.s. The allocation to the groups was randomized, done according to the order of admission to our department. The group one was created by first 80 patients admitted to our department to diagnose and treat tinnitus. Group two was created by the following 40 patients. In order to decrease potential heterogeneity of the groups only the patients with tinnitus duration longer than 1 year, as well as with accompanying hearing loss, were qualified to present research [19].

Before the beginning of the therapy, we conducted the ENT examination, hearing tests (pure tone audiometry, speech audiometry, impedance audiometry, auditory brainstem responses, otoacoustic emissions) and radiological diagnostics—if necessary (head and cervical spine computer tomography/nuclear magnetic resonance). Pathology in the external and/or the middle ear was an excluding criterion. Patients who reported tinnitus in the head, not in ears, or their tinnitus lasted less than 1 year (to minimize the potential rate of spontaneous disappearance), were also disqualified from the research. The patients completed questionnaires (designed by the authors based on the Tinnitus Handicap Inventory) involving 20 questions concerning tinnitus. Possible answers were ‘yes’ obtaining two points, ‘sometimes’, one point, and ‘no’, 0 point. The maximum score was 40, which meant that tinnitus is an enormous problem. On the other hand, receiving 0 points meant that tinnitus is not a disturbing ailment. The person conducting hearing evaluation and administering the questionnaires was unaware of the fact that e.s. or placebo e.s. had been provided.

E.s. was performed with the use of a custom-made apparatus supplied with four batteries of 1.5 V. The device has an on/off button, frequency and current intensity buttons. The external ear canal was filled with 0.9 % saline solution. The active, silver probe was immersed inside external ear canal, avoiding contact with the skin of the canal. The passive electrode was placed on the forehead after skin abrasion with a suitable sterile abrasive electrode paste and clean gauze. The two electrodes were placed to obtain the transmission of the current throughout the hypothetical plane (longitudinal axis) of cochlea. Direct rectangular, positive polarization current was applied via the active electrode. The frequency of the current was equal to the frequency of the rectangular impulse. The duration of the rectangular pulse (the period) depended on the frequency. For 250 Hz, one period lasted 4 min (2-min pulse and 2-min pause). The voltage was constant and equals 3 V. The intensity ranged from 0.15 to 1.15 mA and was applied according to patient’s sensation. The stimulation was started using the maximal intensity of current (1.15 mA); if it was well tolerated, stimulation was continued. However, if the patient reported to feel the pain or other unpleasant sensation, the intensity of the current was decreased to the moment when this feeling stopped. The frequency of the current ranged from 250 to 8,000 Hz and was adjusted according to the tinnitus frequency, so that the frequency of the current and the tinnitus frequency were similar (±1,000 Hz)—selective e.s. Pitch-match frequency was performed for all patients before the beginning of the treatment. The comparison sounds were presented to contralateral ear. The patient was asked to identify the tones at the narrowest noticeable frequency intervals. Single e.s. lasted 4 min. The treatment involved 15 applications of e.s. administered three or four times a week (whole treatment lasted approximately 30 days). In group two, the patients were subjected to similar procedure of e.s. as patients from group one; however, no current was delivered through the electrode, dipped in external acoustic canal. Apart from that, the treatment protocol was the same in both—e.s. (1) and placebo e.s. (2) group. Evaluation of tinnitus and hearing (in pure tone audiometry) was conducted before, directly after, 30 and 90 days after 15 applications of e.s. in groups one and two. Subjective assessment of the results considered a case history and the questionnaire. Change from permanent tinnitus (when patient reported to hear it every day, all the day) to temporary tinnitus (when appeared temporarily or the patient reported to have some periods without tinnitus) was considered an improvement. Regarding questionnaires, the increase in the total points (by at least 20 %) was considered deterioration, whereas their decrease an improvement.

Statistical testing for dependent (correlated) observations, we used the Student’s *t* test for correlated samples (in case of both normal distribution samples) or the Wilcoxon test (when at least one sample had non-normal distribution), for independent (uncorrelated) observations the Mann–Whitney *U* test (when at least one sample had non-normal distribution). In case of nominal features, we used the *λ* ^2^ test (for uncorrelated samples) or the McNemar’s *λ* ^2^ test (for correlated samples). The results of the statistical testing were given as a *p* value (*p* < *p*_max_, e.g. *p* < 0.05 indicated the statistically significant relation on a given level). The *p* > 0.05 referred to the non-significant relation.

The research was approved by institutional review board of Medical University of Lodz (RNN/251/05/KB). All patients gave their written, informed consent prior to inclusion in the study.

## Results

Average duration of tinnitus was similar in both groups (no statistically significant differences *p* > 0.05)—in group one 4.24 years ± 5.29, in group two 3.98 years ± 4.17. The minimal tinnitus duration in both groups was 1 year, the maximal in group one—30 years, in group two—27 years.

Before the treatment, group one (*n* = 119 ears) comprised 106 ears (89.1 %) with permanent and 13 ears (10.9 %) with temporary tinnitus; group two (*n* = 65 ears), 56 ears (86.1 %) with permanent and 9 ears (13.9 %) with temporary tinnitus. In groups one and two, directly after the treatment, the number of ears with permanent tinnitus decreased considerably (*p* < 0.05), group one comprised 58 ears (48.8 %) with permanent and 21 ears (17.6 %) with temporary tinnitus, in 40 ears (33.6 %) tinnitus disappeared; group two 46 ears (70.8 %) with permanent and 15 ears (23.1 %) with temporary tinnitus, in four ears (6.1 %) tinnitus disappeared. After 30 days, statistically significant changes were observed in group one (*p* < 0.05), which were comparable with results returned 90 days later (*p* > 0.05). Changes in group two (after 30 and 90 days) were not significant (*p* > 0.05). When compared, there were apparent differences between results of the groups (*p* < 0.05) (Table 1).

**Table 1:** The evaluation of the treatment considering tinnitus character

Group/*n* (%) ears	Permanent	Temporary	Disappearance	*p*
Group one (*n* = 119 ears)				
Before e.s.	106 (89.1 %)	13 (10.9 %)	–	
Directly after e.s.	58 (48.8 %)	21 (17.6 %)	40 (33.6 %)	<0.05
30 days after e.s.	58 (48.8 %)	40 (33.6 %)	21 (17.6 %)	<0.05
90 days after e.s.	58 (48.8 %)	47 (39.4 %)	14 (11.8 %)	>0.05
Group two (*n* = 65 ears)				
Before placebo e.s.	56 (86.1 %)	9 (13.9 %)	–	
Directly after placebo e.s.	46 (70.8 %)	15 (23.1 %)	4 (6.1 %)	<0.05
30 days after placebo e.s.	50 (77 %)	12 (18.4 %)	3 (4.6 %)	>0.05
90 days after placebo e.s.	48 (73.9 %)	12 (18.4 %)	5 (7.7 %)	>0.05

*p* < 0.05, change statistically significant

*p* > 0.05, change statistically not significant

*e.s*. electrical stimulation

Analysis of questionnaires, directly after the treatment, in group one showed improvement in 45 ears (37.8 %) and in group two, in 20 ears (30.8 %). In group one, 30 days after the last e.s., the statistical analysis showed subsequent improvement (*p* < 0.05) to 51.3 %. On comparing results to the analysis conducted 90 days after the treatment, there were statistically significant differences in groups one and two (Table 2).

**Table 2:** The evaluation of tinnitus treatment based on the questionnaires

Group/*n* (%) ears	Improvement	No changes	Deterioration	*p*
Group one (*n* = 119 ears)				
Directly after e.s.	45 (37.8 %)	65 (54.6 %)	9 (7.6 %)	–
30 days after e.s.	61 (51.3 %)	50 (42 %)	8 (6.7 %)	<0.05
90 days after e.s.	56 (47.1 %)	49 (41.2 %)	14 (11.7 %)	>0.05
Group two (*n* = 65 ears)				
Directly after placebo e.s.	20 (30.8 %)	44 (67.7 %)	1 (1.5 %)	–
3,170 days after placebo e.s.	19 (29.2 %)	43 (66.2 %)	3 (4.6 %)	<0.05
90 days after placebo e.s.	17 (26.1 %)	44 (67.7 %)	4 (6.2 %)	>0.05

*p* < 0.05, change statistically significant

*p* > 0.05, change statistically not significant

*e.s.* electrical stimulation

We recognized subjective and audiometric improvements of hearing (in pure tone audiometry) in group one, at the end of the control period. After the treatment, patients reported subjectively improved hearing in group one in 36 ears (30.2 %), in group two in 14 ears (21.5 %). No deterioration of hearing was reported. In audiometric evaluation after the cycle of e.s. in group one, statistically significant improvement of hearing was registered: for frequencies between 1,000 and 4,000 Hz (by on average 4.35 dB). There was no statistically significant deterioration of hearing in both groups. At the end of the control period, the improvement of hearing remained constant in group one (Table 3).

**Table 3:** The average hearing level (dBHL) in pure tone audiometry

Average hearing level (dBHL)	Frequency (Hz)								
	125	250	500	1,000	2,000	3,000	4,000	6,000	8,000
Group one (*n* = 119 ears)									
Before e.s.	25.6	27	26.4	31.4	35.2	41.1	47.6	56.4	51.6
Directly after e.s.	29.4	28.5	27.5	27.2	30.1	36.5	42.0	53.4	49.6
90 days after e.s.	30.3	29.1	27.3	28.3	31.0	36.9	42.5	53.3	49.4
Group two (*n* = 65 ears)									
Before placebo e.s	30.9	31.4	31.7	33.2	35	39.1	42.0	50.0	49.4
Directly after placebo e.s.	27.9	29.1	31.1	31.5	33.2	35.3	41.5	47.0	45.3
90 days after placebo e.s.	25.6	25.6	27.8	33.3	36.1	35.0	39.5	47.8	47.8

*e.s.* electrical stimulation

## Discussion

The beginning of e.s. clinical application to the hearing organ appeared after observation of tinnitus disappearing after the implantation of a single-electrode cochlear implant. In 1973, House (The House Ear Institute) reported total disappearing of tinnitus after the implantation of the single-electrode cochlear implant (using electrical current to stimulate auditory nerve). Such an effect was noticed later by other authors [20]. This fact was a fundamental observation which resulted in the idea of suppressing tinnitus with electrical current. In this way the idea of the e.s. in tinnitus treatment appeared. Tinnitus suppression was obtained when cochlea, round window, promontorium, preauricular skin or mastoid was subjected to stimulation. In 1974, House suggested a method of evaluation of the hypothetical benefit of the cochlear implantation based on the transtympanal e.s. of the promontory. In case of a sound sensation (reported by a patient during the stimulation), peripheral impairment of the hearing organ (outer, inner hair cells) was considered, whereas when the patient reported hearing no noise central. Similar research was conducted by Bochenek et al. [21]. During e.s. in more than half of the patients, a sound sensation was observed. The authors suggested that in such cases, clinical diagnosis indicated central VIII-th nerve dysfunction, rather than the peripheral one. They admitted, however, that some VIII-th nerve’s fibers, which were not impaired, could have been enough to evoke sound.

Skarzynski et al. [23] and Bochenek et al. [22] proved the usefulness of non-invasive alternative extratympanic ear canal e.s., as a test in prediction of post-operative profits before cochlear implantation. The authors observed reception of the tones as well as speech signal, by some completely deaf patients in whom e. s. via external auditory canal (with a ball-shaped electrode dipped in saline solution) was conducted. In this way, they claim to stimulate the fibers of the auditory nerve, obtaining hearing sensation as an evidence. Despite numerous researches on tinnitus disappearance after cochlear implantation, its mechanism seems to remain unexplained conclusively. As many patients benefit from hearing aid (experiencing tinnitus suppression) we may suspect that the enhancement of the signal in the auditory pathway is the factor responsible for this phenomenon [24]. Sensorineural hearing loss is one of the most apparent risk factor for tinnitus, probably resulting from maladaptive attempts at cortical reorganization due to peripheral deafferentation [25]. As in patients with tinnitus and single-sided deafness (SSD) therapies based on acoustic input (retraining, masking) are impossible, the restoration of peripheral sensory input may be a method of masking/relieving tinnitus. There are some data showing good effects of binaural integration of acoustic (unilateral normal hearing) and electric stimulation (via cochlear implant), which appeared to be superior to the alternative rehabilitation methods of SSD and tinnitus [bone-anchored hearing aid (BAHA), contralateral routing of signal (CROS)] [26]. Although groups of patients implanted with tinnitus and SSD were not numerous, there were studies demonstrating significant improvement reaching 100 % [27–29]. In effect, SSD with severe tinnitus is considered a new indication for cochlear implantation; however, appropriate patients selection is required [27, 29, 30]. Arts et al. [31] state that cochlear implant should be considered as a treatment for tinnitus resulting from SSD (from peripheral-cochlear deafferentation). Furthermore, there may be some predictors of the degree of improvement after such procedure. Song et al. [32] collecting quantitative electroencephalography found positive correlation between increased activity of auditory posterior cingulate cortex and dorsolateral prefrontal cortex and slight tinnitus reduction after cochlear implantation.

In the research, the application of the current was based on our experience. The intensity of current was applied according to patient’s sensation and tolerance, whereas the frequency according to tinnitus pitch (frequency)—selective e.s. As in the majority of cases, the cochlea may be a trigger or ignition site for subjective tinnitus, authors adopted the theory that the frequency of tinnitus might be consistent with the area of damaged outer hair cells in the basilar membrane [33, 34]. For that reason those parameters were similar as well as the tinnitus matching (pitch) method was used in the research. It was done based on ‘Adaptive Method’ according to Tyler’s indications [35, 36]. Despite the fact that psychoacoustic measurements may not correspond to tinnitus severity, such quantification is needed in clinical trials for evaluation of treatment, but it requires standardization of techniques.

Tyler et al. [6] summarize the state of the art knowledge of extra- and intracochlear e.s. in tinnitus. The authors state that the optimal parameters of stimulation are likely different for different subjects. Offut claims that auditory stimulation with specific frequencies within the area of loss of hearing in pure tone audiometry can reduce tinnitus, by suppressing the inner hair cells [37]. However, Dauman et al. [38], using cochlear implant for e.s. of hearing organ, observed that the effectiveness of the stimulation depended on the stimulating frequency and was optimal using 125 Hz. Morawiec-Bajda et al. [15] performed e.s. via external auditory meatus with the active electrode placed on tympanic membrane and the other on the forehead. The improvement was obtained in 46.6 % of cases. Furthermore, using for stimulation frequencies close to tinnitus frequencies, an increase in otoacoustic emission’s amplitude (more distinct in DPOAE than TEOAE) was obtained, as well as increase in amplitude and shortened latencies in auditory brainstem responses.

The theories on the cochlea as an ignition site for tinnitus, together with hypothesized ways of influencing its structures by e.s., may indicate the need for individually modified parameters of e.s [6]. The promontory stimulations were conducted by Aran and Cazals [39] who achieved satisfactory effects (complete or partial improvement of tinnitus condition) in 43 % of cases, compared with 60 % of improvement cases when oval window was stimulated. Ito and Sakakihara [40] via stimulation of cochlea directly with cochlear implant received better outcomes (77 %) than via stimulation of promontory (69 %). The results of invasive (direct) transtympanal e.s. are better when compared with that of non-invasive methods.

As far as an improvement is concerned, our outcomes are comparable to the results of non-invasive e.s. conducted by other authors. The total number of tinnitus disappearance is more apparent in presented research; however, the tendency to decrease with time is observed. Application of hydrotransmissive method in our research greatly simplifies the technique of e.s. This non-invasive procedure allows the doctor to perform it in any outpatient clinics, and as a result, the patient does not have to stay under the medical observation directly after stimulation. Furthermore, hydrotransmissive stimulation allows application of cycle of such stimulations, improving the chances of relieving tinnitus, as well as helping to boost and maintain the improvement of hearing. Recently, a range of device delivering transcutaneous mastoid e.s. has been constructed. The idea was to stimulate in a simple, non-invasive way, giving the patient possibility to perform ‘self’-stimulations at home. However, most reports do not support efficient success rates.

Subsequent improvement of the results after a month is a fact worth noticing. The number of stimulation applications (15), performed regularly, might have had an influence on the subsequent improvement together with its stabilization. In our research, in both treated groups, we observed the change in the nature of tinnitus (permanent to temporary). The change was most noticeable in group one (treated with e.s.)—the number of cases with permanent tinnitus decreased by around 50 %, but in both stimulated groups (groups one and two), the improvement was statistically significant (*p* < 0.05). As patients referred, the change from permanent to temporary tinnitus was meaningful for them, and it allowed to experience some silent periods (often after months or years of presence of continuous, chronic noise in the ear) again. That is why most of the patients considered it an apparent improvement. In literature, such evaluation of the tinnitus treatment has not been found.

In one of the earliest publications concerning e.s. in the tinnitus treatment, Portman et al. [10] described the dependence of the result of stimulation on the polarization of the current. Using direct negative current, a sound sensation was evoked, proving that the VIII-th nerve was stimulated. In case of positive polarization suppression of tinnitus was observed, but only for the time of stimulation. After the procedure was finished, tinnitus appeared again [41]. In our research, in most cases a sound perception was observed during stimulation with positive current, so it may be possible that the factor responsible for such auditory reception is the condition of the hearing organ rather than current polarization. At early studies conducted by Portmann et al. [41] and Aran et al. [42], direct current appeared to be more harmful to the inner ear (than alternating); however, it was more efficient in suppressing tinnitus.

Konopka [43] in his study on the influence of direct current on the hearing organ of guinea pigs demonstrated no pathologic effect on auditory pathway based on the evaluation of summation potentials, cochlear microphonics and auditory brainstem potentials. In present research, we did not notice any destructive influence of direct current based on the hearing evaluation, but in the literature there are some reports on damaging effect of direct current on the cochlea [41, 42]. Furthermore, in our research, pure tone audiometry revealed statistically significant improvement in hearing threshold (*p* < 0.05) in group one, which can mean hypothetically that the function of outer hair cells improved (but in present study, objective measurements such as otoacoustic emissions or auditory brainstem responses were not performed after the treatment). Subjective evaluation of hearing in pure tone audiometry revealed the best effect in group one. Although the degree of the hearing improvement seems not to be clinically meaningful, the hearing level reminded stable and this measurement was repeatable during control period. This might also be the result of disappearance or decreasing tinnitus severity, which could have had a masking effect. Having in mind that sensorineural hearing loss is difficult to treat, especially in chronic course, this improvement appeared more significant, nevertheless it needs further confirmation in objective measurements. In group one we observed a perception of sound in most ears while using direct positive current, with its frequency adopted according to the tinnitus frequency parameters (selective e.s.). In the majority of cases those frequencies corresponded with impaired frequencies in pure tone audiometry—peripheral cochlear impairment. This observation might be coherent with a hypothesis that the sound perception during e.s. proves normal functioning of the auditory nerve.

Many authors highlight the need for placebo-controlled studies to assess placebo effect in tinnitus treatment. The placebo effect is known to cause neurobiological changes comparable to those resulting from pharmacotherapies; however, its mechanisms are not fully understood. According to Benedetti et al. [44] placebo responses can be attributed to the two phenomena: conditioning and expectation of therapeutic benefit. The authors state that expectation can best stimulate a placebo response. Neuroimaging [positron emission tomography (PET) and functional magnetic resonance imaging (fMRI)] and neurotransmitter release measurement have contributed to explanation of some placebo underlying mechanisms. The differences of the placebo effect in patients may reflect variations in the activity in some neurotransmitter systems (dopamine, serotonin, cholecystokinin, opioid). Neuroimaging of placebo analgesia pointed to decreased activity in thalamus, insula and somatosensory cortex [45].

With respect to tinnitus, the placebo effect was mainly assessed in pharmacotherapy (dexamethasone, lidocaine, paroxetine, betahistine, vasodilators, diuretics) and complementary medicine therapies (ginko biloba, acupuncture, massage, meditation) showing no advantage of therapy over placebo [3]. The comparison of transcutaneous electrical stimulation and placebo stimulation resulted in similar outcomes [46, 47]. Duckert [48] assessed placebo effect in tinnitus after saline solution injection instead of lidocain. He obtained improvement in 40 % of tinnitus concluding that each uncontrolled clinical trial may by biased by placebo effect. McFerran and Phillips [3] point to difficulties in evaluating different tinnitus treatment methods as they lack blinding. They cite the Duckert’s experiment (aforementioned) stressing that placebo effect “is often regarded pejoratively by practitioners of modern medicine, overlooking the fact that a placebo is not the same as no effect”. Kapkin et al. [49] assessed placebo effect in transcutaneous e.s. of preaurical region. The rate of improvement in e.s. group was 42.8 % and in placebo group 28.5 %. The rate of worsening was respectively: 16.6 and 42.8 %. The authors used lidocain to deliver local infiltration anesthesia to the stimulated region of the preaurical skin to create identical circumstances during e.s. and placebo e.s. procedure. According to them, substantial difficulty in placebo-controlled study is to establish comparable conditions for treated and placebo group. In case of e.s. the patient will feel the electric current. On the basis of our research and previous experiences only 26 % (31 ears) felt electric current during e.s. In those cases patients reported sensation of pricking, tingling, warming and pain. All patients were informed that during e.s. they can experience some sensations like above, but not necessarily.

According to Hoare et al. [50] most research on tinnitus lack blinding, thus those therapeutic methods remain to be demonstrated conclusively especially those most commonly used: hearing aids, maskers, tinnitus retraining therapy. On the other hand many patients with ear problems/diseases suffer from tinnitus as well, but only in few cases underlying etiology can by clarified. For that reason each therapy, also symptomatic, may be effective especially for those with constant, severe tinnitus.

## Conclusions

1. The application of direct current electrical stimulation of the hearing organ, with current frequencies similar to tinnitus frequencies (selective electrical stimulation), was an efficient method in severe tinnitus treatment.
2. We did not observed a harmful effect of direct current on hearing organ.
